# Psychological resilience among informal care partners of individuals with mild cognitive impairment

**DOI:** 10.1371/journal.pone.0362079

**Published:** 2026-11-13

**Authors:** Alberto Villarejo-Galende, Elena García-Arcelay, Mariló Almagro, Mircea Balasa, Gerad Piñol-Ripoll, Lamberto Landete, Inmaculada Abellan, Angel Berbel, Beatriz Espejo, Miquel Baquero, Juan Marin, Emilio Franco-Macias, Félix Viñuela Fernández, Inmaculada Feria-Vilar, Carmen Perez-Vieitez, Norberto Rodríguez-Espinosa, Albert Puig-Pijoan, Eduard Bargay Pizarro, Eloy Rodríguez-Rodríguez, Jesús Rodrigo, Mercè Boada, Pascual Sánchez-Juan, Sagrario Manzano, Jorge Maurino

**Affiliations:** 1 Department of Neurology, Hospital Universitario Doce de Octubre, Instituto de Investigación Imas12, Universidad Complutense de Madrid, Madrid, Spain; 2 Medical Department, Roche Farma, Madrid, Spain; 3 Confederación Española de Alzheimer y otras Demencias (CEAFA), Madrid, Spain; 4 Alzheimer´s Disease and Other Cognitive Disorders Unit, Hospital Clínic, IDIBAPS, Barcelona, Spain; 5 Cognitive Disorders Unit, Hospital Universitari Santa Maria de Lleida, IRBLLeida, Lleida, Spain; 6 Department of Neurology, Hospital Universitario Dr. Peset, Valencia, Spain; 7 Neurology and Dementia Unit, Hospital San Vicente del Raspeig, Alicante, Spain; 8 Department of Neurology, Hospital de la Cruz Roja, Madrid, Spain; 9 Cognitive-Behavioral Neurology Unit, Hospital Clínico San Cecilio, Granada, Spain; 10 Alzheimer´s Disease Research Group, Hospital Universitari i Politècnic La Fe, IIS La Fe, Valencia, Spain; 11 Dementia Unit, Hospital Clínico Universitario Virgen de la Arrixaca, Murcia, Spain; 12 Dementia Unit, Department of Neurology and Neurophysiology, Hospital Universitario Virgen del Rocío, Universidad de Sevilla, IBiS, Sevilla, Spain; 13 Instituto Neurológico Andaluz, Hospital Victoria Eugenia, Unidad Deterioro Cognitivo, Hospital Virgen Macarena, Universidad de Sevilla, Sevilla, Spain; 14 Department of Neurology, Complejo Hospitalario Universitario de Albacete, Albacete, Spain; 15 Department of Neurology, Hospital Universitario de Gran Canaria Doctor Negrín, Las Palmas de Gran Canaria, Spain; 16 Department of Neurology, Hospital Universitario Nuestra Señora de Candelaria, Santa Cruz de Tenerife, Spain; 17 Unitat de Cognició i Conducta, Hospital del Mar, IMIM, Barcelona, Spain; 18 Department of Neurology, Hospital Universitario Son Espases, Mallorca, Spain; 19 Department of Neurology, Hospital Universitario Marqués de Valdecilla, University of Cantabria, IDIVAL, Santander, Spain; 20 Ace Alzheimer Center Barcelona, Universitat Internacional de Catalunya, CIBERNED, Barcelona, Spain; 21 Fundación CIEN (Centro de Investigación de Enfermedades Neurológicas), Madrid, Spain; 22 Department of Neurology, Hospital Universitario Infanta Leonor, Madrid, Spain; Nathan S Kline Institute, UNITED STATES OF AMERICA

## Abstract

**Background:**

Care partners of individuals with mild cognitive impairment (MCI) encounter unique psychosocial and operational challenges. While dementia research traditionally focuses on advanced cognitive decline, the factors underlying positive psychological adaptation during the early MCI window remain poorly characterized. This study aimed to evaluate the prevalence of psychological resilience among informal care partners of individuals with MCI and identify independent multivariable correlates of high adaptive capacity.

**Methods:**

We conducted a multicenter, cross-sectional study across 19 specialized memory clinics in Spain in collaboration with the Spanish Confederation of Alzheimer's Disease and Other Dementias (CEAFA). Psychological resilience was quantified using the 14-Item Resilience Scale (RS-14). Secondary outcomes included health-related quality of life, caregiver strain, psychological distress, dyadic relationship quality, and perceived affiliate stigma. Multivariable logistic regression was employed to identify independent predictors of moderate-to-high resilience (RS-14 score >81).

**Results:**

We analyzed 196 care partners (mean age: 63.5 [SD 13.1] years, 62.8% women). Overall, 63.3% (n = 124) demonstrated moderate-to-high resilience. In bivariate analyses, higher resilience correlated with superior quality of life, greater sense of competence, and enhanced relationship quality between care partners and care recipients. In multivariable logistic regression, only robust perceived social support (OR=2.37, 95% CI 1.78–3.14, p < 0.001) and lower affiliate stigma (OR=0.09, 95% CI 0.03–0.23, p < 0.001) remained independently associated with moderate-to-high resilience (model fit: Hosmer- Lemeshow *x*^*2*^  = 5.039, *p* = 0.753; Nagelkerke *R*^*2*^ = 0.319).

**Conclusions:**

Psychological resilience strongly aligns with care partner well-being prior to the onset of overt care-recipient dependency. Early psychosocial interventions designed to reinforce functional social support networks and mitigate internalized stigma may offer a valuable framework for supporting care partner well-being.

## Introduction

Mild cognitive impairment (MCI) represents a critical clinical and psychological boundary, serving as a transitional phase between the cognitive decline associated with normative aging and the onset of clinical dementia [1–3]. This syndrome is characterized by objective cognitive deficits that exceed chronological and educational expectations without entirely compromising independence in basic daily self-care [1,2]. Affecting an estimated 10% to 20% of adults aged 65 and older, the unpredictable clinical trajectory of MCI carries an annual dementia conversion rate of 10% to 15%, which imposes a profound emotional burden on affected individuals and their families [3–6].

Consequently, the responsibility of managing these subtle shifts falls heavily on family members, who assume the collaborative role of unpaid informal care partners [6,7]. Dealing with this early cognitive and behavioral transformations introduces a unique trajectory of psychological strain. Unlike the explicit, highly visible care demands of advanced dementia, supporting an individual with MCI involves an intricate layer of invisible labor [7–11]. This dynamic demands constant vigilance to manage understated behavioral shifts, such as apathy, anxiety, and mood lability, long before physical dependency occurs. Beyond this crucial emotional scaffolding, care partners must absorb growing operational responsibilities, including financial oversight, medical supervision, and care coordination, while frequently balancing their own professional workloads [8–11].

Despite escalating daily strain during these early stages of cognitive decline, a subset of care partners maintain high psychological well-being and exceptional adaptive capacities [12,13]. This capacity for positive adaptation amidst persistent adversity is conceptualized as psychosocial resilience [14–16]. Rather than a static personality trait, resilience operates as a dynamic, active self-regulatory process involving the flexible mobilization of emotional, cognitive, and behavioral resources to balance demanding circumstances. Historically, clinical research has focused predominantly on the deficits, stress, and subjective burden associated with caregiving. However, the American Psychological Association guidelines explicitly recommend that clinical investigators look beyond pathology to address the positive aspects of caregiving [17]. Evaluating resilience as a primary protective factor aligns with this mandate, providing an empirical rationale for shifting toward a strengths-based care infrastructure.

While an extensive body of literature has documented the protective role of resilience in late-stage dementia, empirical observations focused specifically on the unique clinical window of MCI remain limited [18,19]. The MCI phase represents a fundamentally distinct paradigm of support. Because care recipients remain superficially autonomous, care partners must continuously navigate ambiguous “gray areas” of independence and progressive memory failure. Identifying the specific modifiable factors that bolster or undermine psychological resilience during this precise period is essential for designing early, proactive clinical frameworks [20]. Accordingly, this study aimed to evaluate the prevalence of psychological resilience among informal care partners of individuals living with MCI and to establish the independent psychosocial correlates associated with high adaptive capacity.

## Methods

### Study design and participants

This multicenter, cross-sectional study was conducted across 19 hospital-based memory clinics in Spain in collaboration with the Spanish Confederation of Alzheimer's Disease and Other Dementias (CEAFA). Adult, informal care partners of individuals diagnosed with MCI were identified and recruited by attending neurologists during routine follow-up visits between July 5 and December 11, 2023. Diagnostic confirmation of MCI followed the clinical criteria established by the National Institute on Aging and the Alzheimer's Association (NIA-AA), paired with a Global Deterioration Scale (GDS) score of 3 [21,22]. To meet eligibility requirements, carer partners had to maintain regular and sufficient contact with their care recipients to ensure a comprehensive familiarity with their functional and neurobehavioral status. The research review board of Hospital del Mar in Barcelona, Spain, approved the study protocol, and all participants provided written informed consent prior to enrollment.

### Outcome measures

The primary outcome was psychological resilience, evaluated via the Spanish version of the 14-Item Resilience Scale (RS-14) [23]. The RS-14 utilizes a 7-point Likert-type scale with cumulative scores ranging from 14 to 98 [24,25]. Higher values reflect greater adaptive capacity, and a threshold score of >81 classified participants as possessing moderate-to-high resilience [24].

To evaluate secondary multi-dimensional psychosocial caregiving domains, we administered a comprehensive battery of validated self-report instruments:

. *Health-related quality of life***:** The 20-item Assessment of Quality of Life (AQoL-6D) quantified general well-being [26]. Item responses yield a single summary utility score ranging from −0.04 to 1.00, where 1.00 signifies full health-related quality of life.

. *Caregiving strain:* The 22-item Zarit Burden Interview (ZBI-22) measured subjective care partner burden [27]. Responses are scored on a 5-point Likert-type scale with total scores ranging from 0 to 88. Higher valued denote greater perceived strain.

. *Psychological distress:* The Hospital Anxiety and Depression Scale (HADS) evaluated affective symptoms using two distinct 7-item subscales for anxiety and depression [28]. Subscale scores range from 0 to 21, with scores >8 identifying probable clinical cases.

. *Hopelessness:* The 20-item, true/false Beck Hopelessness Scale (BHS) measured negative expectations about the future [29]. Total scores range from 0 to 20, with higher values reflecting a greater magnitude of pessimism.

. *Perceived affiliate stigma:* The 22-item Affiliate Stigma Scale (ASS) quantified internalized caregiving-related social shame [30]. Total scores range from 0 to 88, and a validated cutoff score >31 indicates a significant perception of stigmatization.

. *Sense of competence:* The 7-item Short Sense of Competence Questionnaire (S-SCQ) assessed care partner confidence and skill [31]. Scores range from 7 to 35, where higher values indicate an elevated self-perceived sense of competence.

. *Relationship quality:* The 14-item Quality of the Caregiver-Patient Relationship (QCPR) Scale evaluated the subjective quality of the dyadic bond [32]. Total scores range from 14 to 70, where higher scores signify a more positive relationship between care partner and care recipient.

. *Perceived social support:* The 11-item Duke-UNC Functional Social Support Questionnaire (FSSQ) evaluated care partner satisfaction with their social network [33]. Total scores range from 11 to 55, with higher markers indicating more robust perceived support.

. *Coping strategies:* The 28-item Brief Coping Orientation to Problems Experienced (Brief COPE) assessed 14 coping mechanisms [34]. Rated on a 4-point Likert scale (1 = “not at all,” 4 = “a lot”), subscales range from 2 to 8, providing insights into adaptive (problem-focused) and maladaptive (avoidant) strategy utilization.

All self-report instruments were completed in-person on digital tablets during the visit, with clinicians available on-site for technical assistance.

### Statistical analyses

Descriptive statistics were calculated for all variables, with continuous data summarized as mean (standard deviation [SD] or median (interquartile range [IQR]), and categorical variables presented as absolute and relative frequencies (%). Associations between RS-14 scores and secondary outcome measures were evaluated using Spearman’s rank correlations coefficients. Bivariate correlations were reported without formal alpha-adjustment to avoid inflating Type II error risks given the conceptual interdependence among psychosocial measures.

Prior to multivariable modeling, candidate predictors were evaluated for multicollinearity using Variance Inflation Factor (VIF) diagnostics. Initial diagnostic testing revealed severe collinearity for the BHS score (VIF = 18.3) and redundant composite sum scores for the FSSQ and ASS (VIF > 3.5). These redundant measures were excluded from entering the candidate predictor pool. In the refined diagnostic set, all retained candidate variables exhibited acceptable collinearity parameters (VIF < 3.5, average VIF < 2.5).

Multivariable logistic regression was conducted to identify independent correlates of moderate-to-high resilience (RS-14 > 81). The initial candidate predictor pool comprised care partner demographics, care recipient clinical characteristics, and all secondary scale scores. Continuous variables were analyzed as continuous scale measures, whereas categorical variables were dummy-coded using explicit reference groups (sex: male; relationship: spouse/partner). Variable selection was performed using an automated forward stepwise logistic regression procedure based on the Wald statistic (*p*-entry <0.05, *p*-exit >0.10). Potential two-way interaction terms (e.g., social support × affiliate stigma) were evaluated in preliminary models and omitted as they did not reach statistical significance. Overall model fit was evaluated using the Hosmer and Lemeshow goodness-of-fit test and Nagelkerke $R^{\text{2}}$. Complete model parameters (B, S.E., Wald, df, p-value, Odds ratio [OR], 95% CI) and score statistics for not retained variables are provided in Supplementary S1 Table in S1 File.

The target sample size of 200 care partners was established a priori based on multicenter-recruitment feasibility and multivariable modeling precision requirements. Assuming a 50%−60% prevalence of moderate-to-high resilience (100–120 cases), a sample size of 200 provided a minimum of 10 events per variable for multivariable logistic regression model with up to 10 candidate predictors, mitigating the risk of model overfitting.

All analyses were conducted using IBM SPSS Statistics for Windows, Version 28.0 (IBM Corp., Armonk, NY, USA).

## Results

Of 200 care partners initially enrolled, four withdrew prior to completing the assessment battery, yielding a final analytical sample of 196 participants (98% completion rate). Care partners had a mean age of 63.5 (SD 13.1) years, 62.8% (n = 123) were women, and the majority were spouses or partners of the care recipient (n = 134, 68.4%). Care recipients had a mean age of 72.9 (7.0) years, a mean Mini-Mental State Examination score of 25.2 (3.2), and a mean disease duration of 2.9 (2.2) years.

The overall mean RS-14 score was 83.3 (11.3), with 63.3% (n = 124) of care partners demonstrating moderate-to-high resilience. Additionally, 39.3% (n = 77) reported perceived affiliate stigma. Table 1 presents the main characteristics of the sample.

**Table 1:** Main characteristics of the participants.

		Total N = 196	Moderate-high resilience N = 124	Rest of the sample N = 72		*p*-value
Age, years, mean (SD)		63.5 (13.1)	64.1 (13.6)	62.6 (12.4)		0.283
Sex, female, n (%)		123 (62.8)	79 (63.7)	44 (61.1)		0.717
Education, years, mean (SD)		13.0 (4.7)	12.9 (4.8)	13.1 (4.4)		0.735
Currently working, n (%)		65 (33.2)	41 (33.1)	24 (33.3)		0.969
S-SCQ score, mean (SD)		26.1 (6.1)	27.3 (5.8)	24.1 (6.1)		<0.001
ZBI-22 score, mean (SD)		22.3 (13.6)	18.7 (11.8)	28.5 (14.4)		<0.001
QCPR score, mean (SD)		55.6 (8.9)	56.6 (8.6)	53.9 (9.1)		0.037
HADS Anxiety score, mean (SD)		7.1 (4.1)	6.2 (4.0)	8.6 (3.8)		<0.001
HADS Depression score, mean (SD)		4.6 (3.4)	4.1 (3.4)	5.5 (3.1)		<0.001
HADS Anxiety score ≥8, n (%)		82 (41.8)	40 (32.3)	42 (58.3)		<0.001
HADS Depression score ≥8, n (%)		37 (18.9)	18 (14.5)	19 (26.4)		0.041
BHS score, mean (SD)		5.5 (3.6)	4.9 (3.3)	6.6 (4.0)		0.003
ASS score, mean (SD)		1.4 (0.3)	1.3 (0.3)	1.6 (0.4)		<0.001
AQol-6D Utility score, mean (SD)		0.7 (0.1)	0.8 (0.1)	0.7 (0.1)		<0.001
FSSQ score, mean (SD)		4.0 (0.7)	4.2 (0.6)	3.7 (0.8)		<0.001
Problem-focused COPE score, mean (SD)		2.7 (0.7)	2.8 (0.7)	2.6 (0.7)		0.030
Emotion-focused COPE score, mean (SD)		2.2 (0.5)	2.2 (0.5)	2.1 (0.5)		0.302
Avoidant COPE score, mean (SD)		1.6 (0.5)	1.5 (0.5)	1.7 (0.5)		0.003
*Care recipient*						
Age, years, mean (SD)		72.9 (7.0)	73.0 (7.1)	72.7 (6.8)		0.417
MMSE score, mean (SD)		25.2 (3.2)	25.2 (3.4)	25.1 (2.8)		0.531
Disease duration, years, mean (SD)		2.9 (2.2)	2.9 (2.4)	2.9 (2.0)		0.509

AQol-6D = Assessment of Quality of Life; ASS = Affiliate Stigma Scale; BHS = Beck Hopelessness Scale; COPE = Brief Coping Orientation to Problems Experienced; FSSQ = Duke-UNC Functional Social Support Questionnaire; HADS = Hospital Anxiety and Depression Scale; MMSE = Mini-Mental State Examination; QCPR = Quality of the Caregiver-Patient Relationship; SD = Standard deviation; S-SCQ = Short Sense of Competence Questionnaire; ZBI-22 = Zarit Burden Interview. ^*^ Values denote item-level mean scores rather than unadjusted scale totals to preserve measurement consistency.

Bivariate comparisons demonstrated that care partners with moderate-to-high resilience reported superior health-related quality of life, an elevated sense of caregiving competence, and higher dyadic relationship quality with care recipients compared to those with lower resilience (Table 1). Furthermore, highly resilient care partners exhibited lower caregiver strain, reduced anxiety and depression, and fewer feelings of hopelessness and affiliate stigma. Regarding adaptive behaviors, resilient individuals reported greater reliance on problem-focused coping strategies and less utilization of maladaptive, avoidant coping mechanisms.

In multivariable logistic regression analysis, robust perceived social support (OR=2.37, 95% CI 1.78–3.14, p < 0.001) and lower affiliate stigma (OR=0.09, 95% CI 0.03–0.23, p < 0.001) remained independently associated with moderate-to-high resilience. The final multivariable model demonstrated excellent goodness-of-fit, as indicated by the Hosmer and Lemeshow test (*x*^*2*^  = 5.039, *df* = 8, *p* = 0.753) and a Nagelkerke *R*^*2*^ of 0.319. Full multivariable parameter estimates and diagnostic statistics for non-retained variables are provided in S1 File.

Spearman's rank correlation analysis confirmed that higher resilience scores correlated positively with perceived social support (rho = 0.43, *p* < 0.001), health-related quality of life (rho = 0.39, *p* < 0.001), sense of competence (rho = 0.34, *p* < 0.001), and dyadic relationship quality (rho = 0.22, p = 0.002). Conversely, resilience scores correlated negatively with caregiver strain (rho = −0.38, *p* < 0.001), perceived affiliate stigma (rho = −0.37, *p* < 0.001), depressive symptoms (rho = −0.32, *p* < 0.001), anxiety (rho = −0.32, *p* < 0.001), avoidant coping strategies (rho = −0.26, *p* < 0.001), and feelings of hopelessness (rho = −0.24, *p* < 0.001). The complete correlation matrix is detailed in Table 2.

**Table 2:** Spearman´s correlations between the Resilience 14 Scale and outcome measures.

		rho (*p*-value)
	
S-SCQ score		0.34 (<0.001)
ZBI score		−0.38 (<0.001)
QCPR score		0.22 (0.002)
HADS Anxiety score		−0.32 (<0.001)
HADS Depression score		−0.32 (<0.001)
BHS score		−0.24 (<0.001)
ASS score		−0.37 (<0.001)
AQol-6D Utility score		0.39 (<0.001)
FSSQ score		0.43 (<0.001)
Problem-focused COPE score		0.12 (0.086)
Emotion-focused COPE score		0.02 (0.685)
Avoidant COPE score		−0.26 (<0.001)

AQol-6D = Assessment of Quality of Life; ASS = Affiliate Stigma Scale; BHS = Beck Hopelessness Scale; COPE = Brief Coping Orientation to Problems Experienced; FSSQ = Duke-UNC Functional Social Support Questionnaire; HADS = Hospital Anxiety and Depression Scale; QCPR = Quality of the Caregiver-Patient Relationship; rho = Spearman's rank correlation coefficient; S-SCQ = Short Sense of Competence Questionnaire; ZBI = Zarit Burden Interview.

## Discussion

Caring for individuals with MCI presents distinct emotional and practical challenges that frequently elevate care partner strain [6,8–11]. While conventional literature predominantly emphasizes these adverse outcomes, reporting that 20% to 36% of care partners experience clinically significant distress, our findings shift focus toward their inherent capacity for positive adaptation [9]. Specifically, 63.3% of informal care partners in this cohort exhibited moderate-to-high psychological resilience. This observation frames early-stage caregiving not merely as a period of progressive emotional depletion, but as an active window of adaptive capability.

Rather than operating as a static personality trait, psychological resilience functions as a dynamic, self-regulatory process characterized by positive adaptation amidst persistent adversity [35–37]. In this cohort, higher resilience was positively correlated with key psychosocial well-being indicators, including health-related quality of life, an elevated sense of competence, and superior dyadic relationship quality between care partners and care recipients. Conversely, higher resilience correlated inversely with care partner strain, anxiety, depression, hopelessness, and affiliate stigma. These patterns align with broader chronic illness literature, which conceptualizes resilience as a protective psychological buffer that mitigates caregiving-related morbidity and limits reliance on maladaptive coping strategies [38,39].

Our multivariable analysis identified robust perceived social support as a primary independent correlate of moderate-to-high resilience. Although relationship quality showed a positive bivariate correlation with resilience, it was not independently retained in the final multivariable model. During early cognitive decline, an accessible social network mitigates isolation, provides emotional validation, and helps care partners sustain active community engagement [40,41]. Crucially, these findings challenge the assumption that formal support services are unnecessary during early-stage cognitive impairment. Evidence indicates that care partners of individuals with MCI require structured support services, particularly to maintain healthy social networks, at levels comparable to those supporting patients with mild Alzheimer´s disease (AD) [40,42]. Although overall caregiver burden is typically higher in mild AD than in MCI, care partners in both stages face substantial and parallel psychological challenges.

Comparing these two clinical stages reveals that distinct factors drive caregiving strain across the cognitive decline continuum [42]. In MCI, care partner strain is primarily triggered by emerging neuropsychiatric symptoms and memory dysfunction, whereas burden in mild AD stems from a combination of neurobehavioral symptoms and severe functional impairment in instrumental activities of daily living. When care partners perceive their social support networks as functional and accessible, they are better equipped to manage stage-specific stressors and maintain dyadic stability [43].

Equally critical is the independent inverse association observed between perceived affiliate stigma and resilience. In our cohort, nearly 40% of care partners reported significant internalized stigma. Affiliate stigma, whether self-imposed or anticipated from society, erodes personal coping mechanisms, inducing internal shame and creating structural barriers to seeking external care resources [44,45]. The robust negative association in our multivariable model suggests that mitigating internalized stigma is essential for preserving resilience. Public health initiatives aimed at destigmatizing early cognitive impairment are therefore vital [46]. Furthermore, fostering supportive, non-judgmental healthcare environments enables care partners to navigate their evolving roles without adopting protective psychological camouflage, thereby supporting their adaptive capacity.

From a clinical and ethical perspective, comprehensive education, robust social support options, and access to healthcare services should be offered to both patients and care partners immediately upon MCI diagnosis [47]. Actively engaging designated family members or friends respects patient autonomy while enhancing well-being across the care dyad. Implementing early psychoeducation and support programs strengthens mutual concordance, providing a critical foundation for effective, shared coping as cognitive decline progresses.

These findings support shifting clinical practice from isolated patient management toward structured, proactive family support networks immediately following an MCI diagnosis. Specifically, the independent association between social support and resilience suggests that multi-component interventions, combining care coordination, dyadic skills training, and psychoeducation, warrant longitudinal investigation in prospective cohorts [20,48–50].

Several limitations must be considered when interpreting these findings. First, outcome evaluations relied on self-report questionnaires rather than structured clinical diagnostic interviews. Second, demographic parameters such as race and ethnicity were not collected; because the sample was drawn exclusively from specialized hospital-based memory clinics in Spain, cultural nuances regarding familial obligations and stigma perception may limit direct generalizability to other geographical, ethnic, or cultural settings. Third, specific contextual caregiving parameters, namely cohabitation status and total daily or weekly caregiving hours, were not captured in our protocol. Given their established influence on caregiver strain and dyadic dynamics, future studies should systematically record and control for these operational variables. Finally, the cross-sectional design precludes any causal inferences regarding the directional relationships between social support, affiliate stigma, and psychological resilience. Future prospective, longitudinal studies are necessary to determine how these adaptive processes evolve as care recipients transition across clinical stages of cognitive decline.

## Conclusion

This study demonstrates that psychological resilience is significantly associated with the well-being of informal care partners of individuals with MCI, with a substantial majority demonstrating moderate-to-high adaptive capacity. Our findings establish that high resilience is independently associated with robust perceived social support and lower affiliate stigma. Early psychosocial frameworks designed to strengthen functional social support networks and reduce internalized stigma warrant prospective evaluation as potential strategies to sustain adaptive capacity in MCI care partners prior to the onset of severe care-recipient dependency.

## Supporting information

S1 File Multivariable logistic regression model parameters and diagnostics. Table 1: Variables in the equation (final step). Table 2: Variables not in the equation (excluded candidate predictors). Table 3: Model fit and diagnostic statistics.
(PDF)
