# Supplementary material for: Psychological resilience among informal care partners of individuals with mild cognitive impairment
Source: PLoS One. 2026 Nov 13;21(11):e0362079. doi: 10.1371/journal.pone.0362079 (PMC13766236; doi:10.1371/journal.pone.0362079)
Supplement: Supplementary file 1 [file pone.0362079.s001.pdf]

Supporting Information. Full multivariable logistic regression parameters for moderate-to-high resilience

Table 1. Variables in the equation (final step)

Predictor Variable	B	S.E.	Wald	df	*p*-value	OR	95% CI
**FSSQ score**	0.863	0.144	35.944	1	<0.001	2.37	1.78 - 3.14
**ASS score**	-2.364	0.461	26.252	1	<0.001	0.09	0.03 – 0.23
**Constant**	2.052	0.684	9.001	1	0.003	7.78	—

Variable selection performed using forward stepwise logistic regression (Wald statistic; *p*-entry <0.05, *p*-exit >0.10). ASS = Affiliate Stigma Scale; B = unstandardized regression coefficient; CI = confidence interval; df = degrees of freedom; OR = odds ratio; FSSQ = Duke-UNC Functional Social Support Questionnaire; S.E. = standard error; Wald = Wald chi-square statistic.

Table 2. Variables not in the equation (excluded candidate predictors at final step)

Variable Excluded	Score Statistic	df	*p*-value
**AQoL-6D Utility score**	2.057	1	0.151
**ZBI-22 score**	2.175	1	0.140
**S-SCQ score**	0.050	1	0.823
**QCPR score**	0.008	1	0.929
**HADS Anxiety score**	2.910	1	0.088
**HADS Depression score**	0.080	1	0.777
**Problem-Focused COPE score**	1.882	1	0.170
**Avoidant COPE score**	1.230	1	0.267
**BHS score**	1.313	1	0.252

AQoL-6D = Assessment of Quality of Life; ASS = Affiliate Stigma Scale; BHS = Beck Hopelessness Scale; HADS = Hospital Anxiety and Depression Scale; QCPR = Quality of the Caregiver-Patient Relationship; S-SCQ = Short Sense of Competence Questionnaire; ZBI-22 = Zarit Burden Interview.

Table 3. Model fit and diagnostic statistics

	**Value**	**Statistical Significance**
**Log-Likelihood (-2 Log Likelihood)**	216.943	—
**Cox & Snell R^2**	0.239	—
**Nagelkerke R^2**	0.319	—
**Omnibus Model Test (χ^2)**	53.384 ($df = 2$)	$p < 0.001$
**Hosmer and Lemeshow Test (χ^2)**	5.039 ($df = 8$)	$p = 0.753$
